# Theoretically redesigning peritoneal dialysis products for sustainability: A life cycle inventory approach

**DOI:** 10.1177/03913988251415097

**Published:** 2026-02-08

**Authors:** James Larkin, Giulia Ligabue, Gaetano Alfano, Rodrigo Martínez Cadenas, Abass Fehintola, Ingeborg Steinbach, Aycan Yasar, Niccolo Morisi, Gabriele Donati, Brett Duane

**Affiliations:** 1 School of Dental, Child and Public Health, Trinity College Dublin, Dublin, Ireland; 2 Nephrology Dialysis and Kidney Transplant Unit, Azienda Ospedaliero Universitaria di Modena, Modena, Italy; 3 Universidad Autonoma de Madrid, Madrid, Spain; 4 Centre for Sustainable Healthcare, Oxford, UK

**Keywords:** Peritoneal dialysis, sustainable healthcare, life cycle assessment, medical device design, carbon footprint, circular economy

## Abstract

Peritoneal dialysis (PD) is a life-sustaining treatment for end-stage kidney disease but contributes significantly to environmental degradation due to its reliance on single-use plastics, energy-intensive manufacturing and high-volume transport. Redesigning PD products for sustainability is increasingly important as healthcare systems seek to reduce their carbon footprint. In this study, ten high-use peritoneal dialysis (PD) products were redesigned using life cycle thinking. Interventions included low-carbon transport (electric vans), renewable energy and improved waste treatment (pyrolysis). Life cycle inventories (LCIs) were modelled in Open Life Cycle Assessment (OpenLCA)and modelled using cradle-to-gate carbon footprints (kg CO₂-eq) to compare redesigned and conventional versions. All redesigned products achieved carbon footprint reductions, with eight showing decreases greater than 40%. The automated PD set and 2 L dialysate bag saw reductions of 63% and 54%, respectively (saving 1.15 and 0.86 kg CO2-eq per item). The APD machine achieved the largest percentage reduction at 87%, primarily driven by the elimination of printed packaging and the use of renewable electricity. Key contributors to emissions savings across products included lower-impact transport, sustainable packaging materials and circular waste strategies. Redesigning PD products using sustainable materials and processes can deliver substantial environmental benefits without compromising functionality. These findings support evidence-based pathways for reducing emissions in kidney care through product innovation and procurement reform.

## Introduction

Peritoneal dialysis (PD) is a widely used renal replacement therapy for patients with end-stage kidney disease (ESKD), yet its environmental impact is substantial due to the large volume of single-use medical products and associated packaging, energy use and waste generation.^1,2^ The healthcare sector as a whole contributes approximately 4%–5% of global greenhouse gas (GHG) emissions,^3^ with dialysis treatments identified as particularly resource-intensive and emission-heavy.^4,5^

Growing global awareness of healthcare’s ecological footprint has spurred efforts to make clinical practices more sustainable. Within nephrology, the field of green dialysis has emerged to assess and mitigate the environmental impacts of dialysis care.

This study aims to redesign 10 core products and their life cycles used in PD to reduce their environmental impact, with a focus on carbon emissions, resource use and end-of-life outcomes. Conducted as part of the KitNewCare project, the goal is to identify the products and processes contributing most significantly to environmental impacts and model interventions which could reduce their negative impact. By doing so, the study supports the development of evidence-based strategies to reduce the ecological burden of PD while upholding high standards of patient care. Data were collected at the Nephrology Dialysis and Kidney Transplant Unit of the AOU Policlinico di Modena in Modena, Italy.

## Literature review

This study focusses on the redesign of ten commonly used PD products, ranging from dialysate bags and drainage systems to disinfectants and medical kits, with the aim of reducing their carbon footprints through sustainable design principles. The approach involves using renewable energy and low-carbon transport, and improving recyclability and waste treatment strategies such as pyrolysis. Although bio-based plastics were evaluated during the modelling phase, they were not adopted due to limited emissions savings and unresolved barriers to real-world use in medical contexts.

Several life cycle assessments (LCAs) have quantified the impacts of PD and haemodialysis, identifying hotspots such as polymer production, transport and waste treatment.^6,7^ Sustainable product design presents a key intervention point to address these hotspots by substituting materials, optimising manufacturing and improving end-of-life options.

This work contributes to the growing body of research advocating for low-carbon, circular healthcare systems. By demonstrating feasible and quantifiable environmental improvements at the product level, it offers practical pathways for manufacturers, healthcare providers and policymakers to reduce the environmental impact of PD care without compromising patient safety or product performance.

This assessment is part of the KitNewCare project, an EU co-funded initiative aimed at making kidney healthcare more environmentally sustainable. KitNewCare focusses on kidney care, an area with significant disease burden and resource footprint, and identifies sustainable solutions to reduce carbon emissions while optimising health outcomes, social equity and costs. Starting with selected Nephrology clinics, including Modena, Italy, the project’s mission is to drive the adoption of sustainable practices across nephrology care.^8^

## Methods

### Study design

This study aimed to redesign ten core products and their life cycles used in PD to reduce their environmental impact, with a focus on carbon emissions, resource use and end-of-life outcomes. A product-level life cycle approach was taken, following ISO 14040 and ISO 14044 guidelines for life cycle assessment (LCA).^9,10^ The study did not conduct full impact assessments for each redesigned product but instead focussed on compiling revised life cycle inventories (LCIs) for more sustainable product alternatives.

### Product scope

The ten products redesigned in this study were:

1. 5 L Dialysate Bag
2. 2 L Dialysate Bag
3. Automated Drainage System
4. Automated PD Set
5. APD Machine
6. 3 L Empty Drainage Bag
7. Drainage Set (tubing and connectors)
8. Hand Washing Gel
9. Disinfectant Spray
10. Medical Kit (gloves, dressings and masks)

### Functional unit

The functional unit for this study is defined as one complete item of each redesigned PD product, representing typical use within the treatment pathway. This unit allows for a consistent comparison of environmental impacts between conventional and redesigned product versions on a per-item basis.

### Redesign strategy

Redesigns were guided by sustainable product design principles, including:

- Material substitution: Recycled cardboard and recycled paper for printed and packaging components.
- Energy sourcing: Use of renewable electricity for manufacturing where feasible.
- Waste reduction: Lighter-weight packaging and recyclable product designs (e.g. instruction leaflet from recycled paper).

### Inventory modelling approach

Each product’s life cycle inventory was developed using:

- Primary data: material identification (e.g. weights, polymers and packaging) from actual products and packaging used in Italian PD practice.
- Secondary data: Ecoinvent v3.1 (cut-off system model) for background processes, including material production, energy use, transport and waste treatment.^11^

Where specific med recycled materials were not present in Ecoinvent, custom unit processes were created using literature-based conversion efficiencies and energy use estimates (e.g. bio-based LDPE from sugarcane ethanol, recycled corrugated cardboard and recycled plastic pallet production).

### Outputs

For each redesigned product, a revised list of inputs and outputs was compiled in OpenLCA based on:

- Material inputs (e.g. plastics, paper and chemicals)
- Energy use (electricity and steam)
- Water consumption
- Emissions (fossil CO2 and wastewater)
- Waste generation (recyclable plastic, general waste and hazardous clinical waste)

These inventories were compared against baseline configurations from existing LCI models to evaluate potential reductions in carbon and material impacts. The complete list of inputs and outputs for each product is available at: James Larkin.^12^

Table 1 outlines the specific sustainability interventions applied to each product, including the use of recycled plastics, low-carbon logistics and circular end-of-life strategies.

**Table 1.** Sustainability interventions applied to peritoneal dialysis products.

Product	Recycled plastics	Recycled packaging	Electric transport	Sustainable manufacture	Waste treatment
5 L dialysate bag	Yes	Yes	Yes	Yes	Yes
2 L dialysate bag	Yes	Yes	Yes	Yes	Yes
Automated drainage system	Yes	Yes	Yes	Yes	Yes
Automated PD set	Yes	Yes	Yes	Yes	Yes
APD machine	Partial	Yes	Yes	Yes	Yes
3 L empty bag	Yes	Yes	Yes	Yes	Yes
Drainage set	Yes	Yes	Yes	Yes	Yes
Hand washing gel	Yes	Yes	Yes	Yes	Yes
Disinfectant	Yes	Yes	Yes	Yes	Yes
Medical kit	Yes	Yes	Yes	Yes	Yes

### Tools

Modelling was conducted using *OpenLCA 1.10.3*,12 with *ecoinvent v3.1* as the background database.^13^ Microsoft Excel was used to prepare inventory data, and visual modelling was supported by schematic process maps for each redesigned item.^14^

### Ethics

Ethical approval was not required for this study, as it focussed exclusively on the environmental assessment of medical products and treatment processes within peritoneal dialysis. The research did not involve human participants, patient data or any activities falling under the scope of institutional review board (IRB) oversight. All data collected related solely to the physical characteristics, material composition and life cycle impacts of medical devices and associated logistics, with no personal or identifiable information involved.

## Results

The environmental performance of the ten redesigned PD products was compared with their conventional counterparts using cradle-to-gate carbon footprint values expressed in kilograms of CO2-equivalent (kg CO2-eq) per item. The redesigned products demonstrated substantial reductions in carbon emissions across nearly all categories.

**Figure 1.**
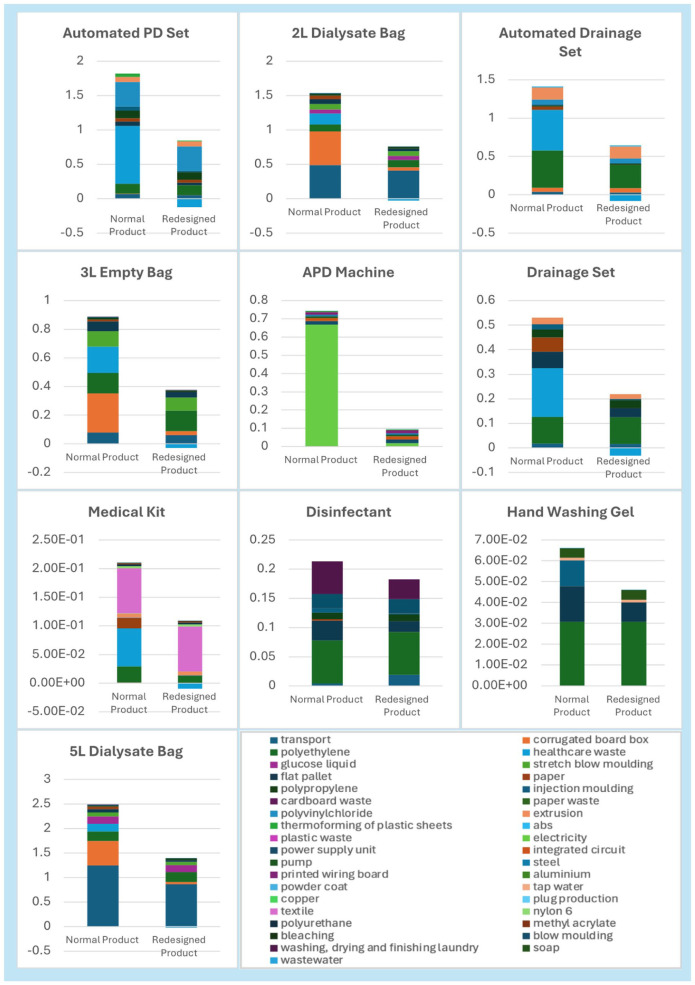
LCA results (kg CO2-eq). PD: peritoneal dialysis; APD: automated peritoneal dialysis.

Contribution Graphs Figure 1 displays the comparison between the normal product and redesigned product, displaying which exact factors contribute to the decrease in carbon footprint.

The largest absolute reduction was observed in the 5 L dialysate bag, the carbon footprint of which decreased from 2.37 to 1.36 kg CO2-eq per item, representing a 42% reduction. This was primarily due to the replacement of virgin corrugated cardboard with recycled board, the use of electric van transport instead of diesel freight, and inclusion of a pyrolysis end-of-life scenario. Similarly, the 2 L dialysate bag achieved a 54% reduction, from 1.59 to 0.73 kg CO2-eq.

The automated PD set, previously one of the most impactful items (1.83 kg CO2-eq), had its carbon footprint reduced to 0.68 kg CO2-eq (63% reduction) through the substitution of virgin corrugated cardboard with recycled board and recycled alternatives, sustainable manufacturing processes using renewable energy and improved end-of-life waste treatment. These items are commonly manufactured using virgin plastics such as polypropylene or polyethylene.

The 3 L empty drainage bag showed a 61% reduction in emissions, from 0.89 to 0.35 kg CO2-eq. Likewise, the footprint of the automated drainage set was reduced from 1.25 to 0.57 kg CO2-eq (54%). These improvements were primarily due to the use of recycled packaging and more sustainable blow moulding using renewable electricity.

The APD machine, a more complex electrical product, saw a dramatic reduction of 87% from 0.75 to 0.10 kg CO2-eq. This was achieved through renewable electricity during the treatment and elimination of unnecessary printed materials and packaging.

The drainage set dropped from 0.54 to 0.20 kg CO2-eq (63%), while the medical kit was reduced by 39%, from 0.25 to 0.15 kg CO2-eq. The hand washing gel and disinfectant both demonstrated significant reductions, dropping by 34% and 6% respectively.

Table 2 presents the percentage reductions achieved for each product through the applied interventions.

**Table 2.** Percentage carbon footprint reductions achieved through product redesign.

Product	Recycled plastics (%)	Recycled packaging (%)	Electric transport (%)	Sustainable manufacture (%)	Waste treatment (%)	Total reduction (%)
5 L dialysate bag	12.8	6.4	10.6	4.3	8.5	42.6
2 L dialysate bag	16.2	8.1	13.5	5.4	10.8	54.1
Automated PD set	18.8	9.4	15.7	6.3	12.6	62.8
APD machine	26	13	21.7	8.7	17.3	86.7
3 L empty bag	18.2	9.1	15.2	6.1	12.1	60.7
Drainage set	18.9	9.4	15.8	6.3	12.6	63
Hand washing gel	10	5	8.3	3.3	6.7	33.3
Disinfectant	1.9	0.9	1.6	0.6	1.3	6.3
Medical kit	12	6	10	4	8	40
Automated drainage system	16.3	8.2	13.6	5.4	10.9	54.4

These were achieved mainly through improvements in packaging and minimised transport emissions.

Overall, eight of the ten products experienced reductions greater than 40%, with most improvements driven by material substitution of packaging, low-carbon transport, renewable energy use and end-of-life waste diversion via pyrolysis. These results support the feasibility of sustainable redesign for common PD products without compromising function.

## Discussion

This study demonstrates that substantial reductions in the environmental impact of PD products can be achieved through targeted product redesign. By integrating recycled materials, using renewable energy sources, switching to electric vehicle transport and implementing improved end-of-life waste treatment methods such as pyrolysis, the carbon footprints of all ten redesigned PD products were notably reduced. These interventions reflect feasible and scalable sustainability strategies in medical device supply chains.

The most significant reductions were observed in high-volume, single-use products such as dialysate bags and components of automated PD systems. These items are packaged with energy-intensive materials like corrugated cardboard boxes and plastic films, making them particularly impactful targets for redesign. Transitioning from conventional lorry and container shipping to electric van transport also contributed meaningfully to emissions reductions. Our results align with studies that highlight energy sources, freight transport and packaging as key environmental hotspots in healthcare supply chains.^15,16^

This policy provides clear, actionable means for manufacturers to reduce their environmental footprint, aligning with emerging requirements from national procurement agencies. As one example, NHS Supply Chain has begun incorporating environmental criteria into tenders, setting a precedent for lifecycle carbon footprint thresholds in public healthcare procurement.^17^ This paper provides additional support for building a framework to guide manufacturers in adapting to these evolving expectations.

Pyrolysis is a process that thermally decomposes plastic waste in the absence of oxygen to generate useful outputs such as fuels, gases or chemical feedstocks. It is adopted in this study as an end-of-life strategy for plastic waste, demonstrating environmental benefits by diverting waste from landfill and incineration and recovering energy or chemical feedstocks. Although still in development, and more open loop focussed (as products can be used to create other different plastics) pyrolysis still aligns with the EU Circular Economy Action Plan and offers an innovative route to enhance waste circularity in medical settings.^18^ Future iterations could also explore closed-loop recycling and producer responsibility schemes to further reduce the environmental footprint of PD components.

While this study primarily focussed on environmental impacts, cost-effectiveness is also a critical area for future exploration. Renewable energy can require infrastructure change, for example, the installation of solar panels, but can also be a change in supplier with cost repercussions.^19^ Sustainable materials may incur higher upfront costs but could lead to savings via reduced waste disposal fees and packaging efficiencies. For example, pyrolysis may offer financial as well as environmental advantages in managing medical waste.^20^

Scalability of these interventions is an important consideration for broader implementation. Although the study’s data collection was based in Italy, the core redesign strategies, such as material substitution, electrified transport and improved waste management, are applicable across healthcare systems. While absolute carbon reductions may differ based on regional factors like energy mix, transport logistics and waste infrastructure, the underlying principles of sustainable product redesign remain consistent and replicable.

Although this study focussed on clinical settings, home-based PD presents additional opportunities and considerations for emission reductions. While the logistics of patient delivery and waste collection are different, existing evidence suggests that variations in waste incineration temperatures between hospital and general waste streams may not result in major differences in carbon emissions. For example, special waste incinerators used in hospitals typically operate between 850°C and 1200°C, while general waste incinerators reach 950°C–1000°C, resulting in relatively comparable thermal profiles.^21,22^ Future research should explore emissions profiles across home and clinic contexts, particularly considering the decentralisation of treatment delivery.

Behavioural aspects such as patient and clinician acceptance also warrant attention. While usability issues were not part of this paper, future co-design with stakeholders could help fine-tune product acceptability and identify potential trade-offs. Research in healthcare waste management suggests that behavioural interventions can enhance waste categorisation, segregation and therefore sustainability.^23^

One of the key barriers to further reducing the environmental burden of PD products lies in the current limitations of bio-based plastics in medical applications. Bio-based high-density polyethylene (bio-HDPE), though environmentally preferable in terms of life-cycle greenhouse gas emissions, cannot yet replace fossil-derived polymers in most medical products. This is due to unmet regulatory requirements for biocompatibility, sterilisation stability and mechanical performance.^24,25^ While bio-HDPE derived from renewable sources such as sugarcane ethanol exists, data on its durability under medical sterilisation and clinical usage is lacking, and its supply chain remains underdeveloped.^26^ Current EU Medical Device Regulation (EU MDR 2017/745) and ISO 10993 standards present additional barriers, particularly for Class II and III medical devices, by requiring extensive biocompatibility testing, sterility validation and long-term safety data that many bio-based plastics have yet to meet. Engaging regulators early in the innovation process is essential to enable approval pathways for sustainable materials.^27^

In addition to regulatory and technical challenges, the sustainability of bio-based plastics also depends critically on the feedstock used. To ensure true environmental benefit, bioplastics should ideally be derived from agricultural waste or non-food biomass rather than crops grown specifically for polymer production. Using first-generation bio-feedstocks such as sugarcane or maize risks indirect land use change, deforestation and competition with food production, potentially leading to greater environmental harm. Thus, future development of bio-based medical plastics should prioritise second-generation feedstocks, such as bagasse, corn stover or other lignocellulosic residues, to avoid trade-offs with land use, biodiversity and food security. A precautionary approach should be taken before recommending wide adoption of bioplastics, ensuring that material substitution does not inadvertently shift environmental burdens elsewhere in the healthcare system.

### Future directions for sustainable PD practice

Digitalisation offers a novel route to reduce environmental impact. Replacing printed instruction leaflets with QR code-based digital resources could reduce paper use and packaging bulk.^28^

Programmes facilitating the collection and recycling of PD materials from patients’ homes have shown promise. For instance, Baxter’s initiative allows patients to recycle plastic and cardboard waste, which is then collected by delivery drivers. Such programmes improve waste management and contribute to sustainability efforts in home dialysis settings.^29^

Implementing incremental PD, where the dialysis dose is gradually increased, can lead to significant environmental benefits. This approach not only lessens environmental impact but also maintains clinical effectiveness.^30^

The adoption of telemedicine in PD care has been associated with reduced carbon emissions. Telehealth not only reduces the need for patient travel but also supports continuous patient monitoring and education.^31^

Transitioning to electric vehicles (EVs) for patient transportation can significantly lower healthcare related carbon emissions. Research indicates that a 30% shift to EVs could reduce healthcare travel emissions to 27.6 Mt CO2e annually. Implementing EVs for routine patient visits and supply deliveries in PD programmes can contribute to environmental sustainability.^32^

Overall, this work supports the transition to sustainable healthcare by offering a replicable model for low-carbon product design in nephrology. It demonstrates how nephrology, through initiatives like KitNewCare, is leading the healthcare sector in sustainable innovation. Further studies should integrate broader environmental impact categories, economic analyses and policy engagement to drive system-wide change.

### Limitations

While this study achieved significant reductions in product-level carbon footprints through redesign, some limitations must be noted. Bio-based plastics, initially considered as substitutes for fossil-derived materials, were ultimately excluded after modelling revealed only marginal environmental improvements and ongoing challenges related to regulatory approval, supply consistency and performance in clinical settings. This highlights a key limitation of current sustainable materials science, where promising alternatives are not yet ready for widespread adoption in healthcare.

Additionally, the study primarily focussed on carbon footprint as the impact metric, omitting other important environmental and social categories such as water use, toxicity or cost. The findings are based on cradle-to-gate approach, which capture use-phase or disposal scenarios beyond modelled end-of-life processes like pyrolysis. Finally, while the redesigns were grounded in practical feasibility, clinical validation of the modified products was beyond the scope of this study and remains a necessary step for implementation.

## Conclusion

This study demonstrates that redesigning PD products using sustainable materials, renewable energy and low-carbon logistics can substantially reduce their environmental impact. Most redesigned items achieved carbon footprint reductions exceeding 40% without compromising functional performance.

These findings offer clear, actionable guidance for manufacturers and healthcare providers aiming to decarbonise medical supply chains. Policymakers are encouraged to support procurement standards that incentivise sustainable design. Future research should build on this work by incorporating full life cycle impact assessments, engaging with clinical stakeholders and evaluating real-world implementation to inform scalable sustainable practices in kidney care.

## Appendix

### Notation

CO2-eq Carbon dioxide equivalent, a standard unit for measuring carbon footprints by converting different greenhouse gases into the amount of CO2 that would have the same global warming potential.

kg Kilogram, a unit of mass.

kg CO2-eq Kilograms of carbon dioxide equivalent, used to express the carbon footprint of materials or processes.

LCA Life Cycle Assessment, a method for evaluating the environmental impacts associated with all stages of a product’s life.

LCI Life Cycle Inventory, a phase of LCA involving data collection of material and energy inputs and outputs for a product or system.

MJ Megajoule, a unit of energy equal to one million joules.

PD Peritoneal dialysis, a treatment for end-stage kidney disease using the peritoneum as a membrane for fluid exchange.

% Percent, a unit expressing a number as a fraction of 100.
